# Comparison of Nanofiltration with Reverse Osmosis in Reclaiming Tertiary Treated Municipal Wastewater for Irrigation Purposes

**DOI:** 10.3390/membranes11010032

**Published:** 2021-01-02

**Authors:** MhdAmmar Hafiz, Alaa H. Hawari, Radwan Alfahel, Mohammad K. Hassan, Ali Altaee

**Affiliations:** 1 Department of Civil and Architectural Engineering, Qatar University, Doha P.O. Box 2713, Qatar; mh1201889@qu.edu.qa (M.H.); ra1404482@qu.edu.qa (R.A.); 2 Center for Advanced Materials, Qatar University, Doha P.O. Box 2713, Qatar; mohamed.hassan@qu.edu.qa; 3 School of Civil and Environmental Engineering, University of Technology in Sydney, 15 Broadway, Ultimo, NSW 2007, Australia; Ali.Altaee@uts.edu.au

**Keywords:** irrigation water, reverse osmosis, nanofiltration, treated sewage effluent, water reuse

## Abstract

This study compares the performance of nanofiltration (NF) and reverse osmosis (RO) for the reclamation of ultrafiltered municipal wastewater for irrigation of food crops. RO and NF technologies were evaluated at different applied pressures; the performance of each technology was evaluated in terms of water flux, recovery rate, specific energy consumption and quality of permeate. It was found that the permeate from the reverse osmosis (RO) process complied with Food and Agriculture Organization (FAO) standards at pressures applied between 10 and 18 bar. At an applied pressure of 20 bar, the permeate quality did not comply with irrigation water standards in terms of chloride, sodium and calcium concentration. It was found that nanofiltration process was not suitable for the reclamation of wastewater as the concentration of chloride, sodium and calcium exceeded the allowable limits at all applied pressures. In the reverse osmosis process, the highest recovery rate was 36%, which was achieved at a pressure of 16 bar. The specific energy consumption at this applied pressure was 0.56 kWh/m^3^. The lowest specific energy of 0.46 kWh/m^3^ was achieved at an applied pressure of 12 bar with a water recovery rate of 32.7%.

## 1. Introduction

Water scarcity is a major challenge that affects food security worldwide, particularly in arid regions. The United Nations (UN) estimates that agriculture accounts for 70% of water usage around the world [1]. Municipal treated wastewater could be an economical solution to be used as irrigation water and a source of nutrients [2]. Treated wastewater can improve soil health and reduce fertilizers consumption. Treated wastewater is rich in pathogens, organics, sodium and chloride; therefore, it could damage soil. The water quality for irrigation water is mainly characterized in terms of total dissolved salts, pH, and different concentrations of ions and cations (e.g., Na, Cl, NO_3_, SO_4_, PO_4_, K, Ca, and Mg). Enhancing the quality of treated wastewater to meet irrigation standards has become a necessary practice. In order to reach the required quality of treated wastewater, membrane technologies are considered to be a critical element.

Shanmuganathan et al. (2015) assessed the performance of nanofiltration (NF) and reverse osmosis (RO) for the treatment of microfiltered municipal wastewater to be used for the irrigation of food crops [2]. The used nanofiltration membranes were NP 010, NP 030 and NTR 729HF with a molecular weight cut-off (MWCO) 1000, 700 and 400, respectively. The reverse osmosis membrane was the seawater reverse osmosis membrane produced by Woongjin Chemical with an MWCO of 100. The used RO membrane must be operated at high applied pressure (i.e., a minimum of 40 bar). Their study focused on the permeate water quality without taking into consideration the high energy consumption of SWRO operated at high pressure. It was found that the water produced using NF and RO alone was not suitable for irrigation for food crops due to poor water quality. However, the hybrid NF-RO process was capable of producing permeate which meets the water quality standards for irrigation of food crops. Li et al. (2016) studied the performance of nanofiltration for the treatment of municipal wastewater using various applied pressures and feed solution pH [3]. It was found that the optimum performance was obtained at an applied pressure of 12 bar, a flow rate of 8 LPM and pH = 4. A pilot-scale study conducted by Oron et al. (2006) showed that by using a hybrid ultrafiltration–reverse osmosis (UF-RO) technology, water suitable for irrigation can be produced from secondary treated municipal wastewater [4]. The cost of the process was between 0.16 and 0.24 USD/m^3^ water. Mrayed et al. (2011) used a hybrid nanofiltration–reverse osmosis (NF-RO) system to produce irrigation water from secondary treated effluent [5]. They used polyacrylic acid (PAA) as a chelating agent. The addition of PAA helped in the formation of covalent bonds among different nutrients in the feed which improved the rejection rate for those nutrients. Egea-Corbacho et al. (2019) tested the performance of a pilot-scale nanofiltration membrane for the treatment of secondary treated wastewater effluent [6]. It was found that the product water quality complies with the Spanish Royal Decree 1620/2007. This was concluded by considering *Escherichia coli*, total suspended solids and turbidity. Still, the authors did not compare the concentration of various elements in the permeate water with allowable limits (i.e., phosphates, nitrates, total dissolved solids, ammonium, sodium and chloride). A study from Chon et al. (2012) used hybrid technology composed of a membrane bioreactor and nanofiltration to produce irrigation water from municipal wastewater [7]. It was found that the physicochemical properties and molecular weight cut-off were the most critical aspects in the removal of nutrients from the water. Gu et al. (2019) evaluated the performance of the trihybrid anaerobic membrane bioreactor (AnMBR)–reverse osmosis (RO) –ion exchange (IE) process for the transformation of microfiltered municipal wastewater to high-grade clean water [8]. The net energy consumption of the process was 1.16 kWh/m^3^, and the product water was found to be suitable for industrial and indirect human applications. Hafiz et al. (2019) used FO to produce irrigation water from treated sewage effluent (TSE) [9]. The feed solution and draw solution for the FO were TSE, and an engineered fertilizing solution (0.5 M NaCl and 0.01 M (NH_4_)_2_HPO_4_), respectively. The draw solution was regenerated using RO. The specific power consumption was between 2.18 and 2.58 kWh/m^3^. Liu et al. (2011) tested NF and RO for the reclamation of textile wastewater in terms of COD rejection and salinity removal [10]. It was found that NF had more severe flux decline compared to RO due to high membrane fouling in the NF process. The total salt rejection of RO was higher than NF. Qi et al. (2020) analyzed the removal efficiency of pollutants in municipal wastewater treatment plants in China along with the operational costs [11]. It was found that biological oxygen demand (BOD_5_) was the highest removed pollutant, while total nitrogen (TN) was the lowest to be removed. It was recommended that higher TN removal efficiencies should be achieved in order to obtain a better effluent quality.

Previous studies assessed the performance of various membrane processes for the treatment of secondary treated wastewater. However, little information is available on the performance of reverse osmosis and nanofiltration to further treat tertiary treated wastewater to generate irrigation water for food crops. This paper is focused on optimizing the performance of the processes to generate a permeate suitable for the irrigation of food crops. The product water quality must comply with the Food and Agriculture Organization (FAO) standards. It is recommended to select a single membrane process that can generate high-quality irrigation water from treated sewage effluent with minimal energy requirements. This study aims to compare the performance of nanofiltration and reverse osmosis for the further treatment of ultrafiltered municipal wastewater for the irrigation of food crops. The performance of each technology was evaluated under different applied pressures in terms of water flux, energy consumption and quality of permeate.

## 2. Materials and Methods

### 2.1. Feedwater

The feed solution used in this study was ultrafiltered tertiary treated sewage effluent (TSE). TSE samples were collected from Doha north wastewater treatment plant. The wastewater treatment plant has three treatment stages: (1) the preliminary treatment that contains step screens and vortex degritters; (2) the secondary treatment stage that contains a bioreactor and clarifier; (3) the tertiary treatment stage that contains a chlorine dosing tank, a multimedia filter, an ultrafiltration membrane process and UV disinfection. In total, 20 L of the treated sewage effluent was collected from the treatment plant twice a week and then stored at 2 °C to preserve the water quality. The characteristics of the TSE sample are shown in Table 1. The maximum limit of the listed parameters was recommended by FAO [11]. The use of this feed water on food crops was unsuitable because of excessive total dissolved solids (TDS) and high ions/cations. The concentration of heavy metals was below the maximum limit recommended by FAO [12]. The conductivity of samples was measured using an OAKTON PCD650 multimeter. Anion concentration was measured by ion chromatography (Metrohm 850 Professional IC) (Metrohm, Herisau, Switzerland), and cation concentration was measured using plasma emission spectroscopy (iCAP 6500-ICP-OES CID) (Thermo Scientific, Waltham, MA, USA). Before measuring the concentration of anions and cations, samples with a conductivity value above 1 mS/cm were diluted using deionized water to a conductivity value below 1 mS/cm. This was carried out to eliminate the interference of high peaks of Na and Cl which may affect the readings of other elements. The turbidity was measured using a turbidity meter (Hach 2100p) (Hach, Dever, CO, USA). Metal concentration was measured using inductively coupled plasma—mass spectrometry (Nexion 300D) (Nexion, Gujarat, India).

### 2.2. Experimental Setup

**Figure 1:**
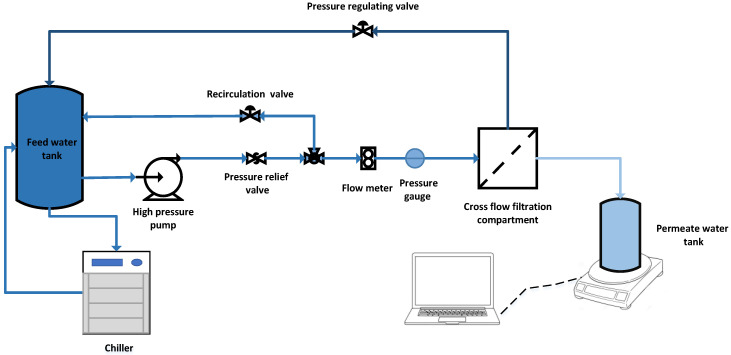
A schematic diagram for the crossflow lab-scale membrane test skid.

A schematic sketch for the bench-scale membrane testing skid is shown in Figure 1. A crossflow CF042D cell made of acetal copolymer provided by Sterlitech-USA was used in the nanofiltration and reverse osmosis processes. The cell dimensions are 12.7 × 8.3 × 10 cm with an active length of 9.2 cm, width of 4.6 cm and 0.23 cm slot depth. Two aluminum tanks were used to store the feed and the permeate water. A HYDRACELL pump (M-03S) (230 V, 50 HZ, 3 PH, 6.7 LPM) was used to pressurize the feed water into the system. A water chiller (PolyScience Chiller) was used to maintain the feedwater temperature at room temperature (25 ± 2 °C). The pressure was regulated through the system using a back pressure control valve. The flow rate of the feed solution was measured using a flow meter (Read Panel Mount Flow Meter) supplied by (Sterlitech, Washington, WA, USA). The permeate flux was measured using a Mettler Toledo—ICS 241 digital balance that was connected to a computer. A specific quantity (3 L) of TSE was used as a feed solution in both processes. The applied pressure in the RO and the NF processes varied between 10 and 20 bar with an increase of 2 bar for each experiment. The flow rate was 3.5 LPM, and the experimental running time was 4 h. The cross-flow velocity in the experiments was kept at 0.55 m/s. The used RO membrane was BW30LE produced by DOW FILMTEC. The used RO membrane is a polyamide thin-film composite (TFC) membrane with a molecular weight cut-off 100 Da. The used NF membrane was NF90 produced by DOW FILMTEC. The used NF membrane is a polyamide TFC membrane with a molecular weight cut-off 200–400 Da. All experiments were repeated three times. and the average of the results is presented. Characteristics for the nanofiltration and reverse osmosis membrane used in the experiment were summarized in Table 2. Three samples (50 mL each) were collected after each experiment for the evaluation of product water quality.

## 3. Results and Discussion

### 3.1. Effect of Feed Pressure on Water Flux and Recovery Rate

**Figure 2:**
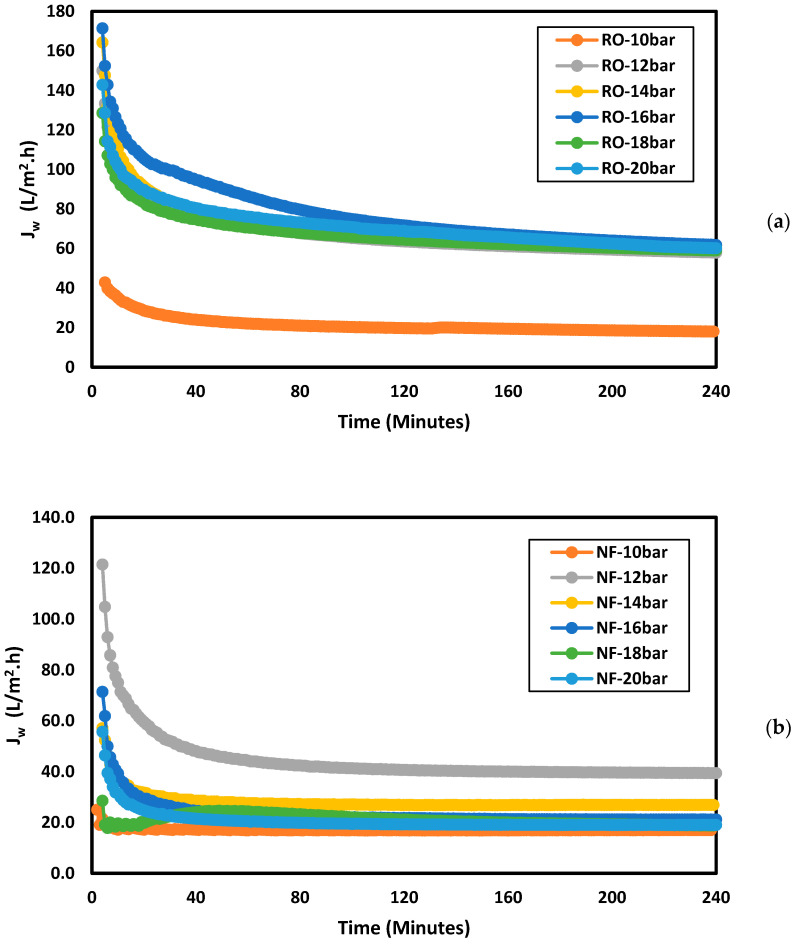
Water flux using treated sewage effluent (TSE) as feed water at different applied pressures in (**a**) reverse osmosis (**b**) nanofiltration.

The water flux $\left(J_{w}\right)$ in the RO process and the NF process was calculated using Equation (1) [16]:

(1) $$J_{w}=\left(\frac{V_{p}}{A_{m}\;\times \;t}\right)$$

where $V_{P}$ is the permeate volume (L),$\;A_{m}$ is the membrane area (m^2^), and *t* is the time of operation (h). Figure 2a,b present the change in water flux in RO and NF with time, respectively. It can be seen from Figure 2a,b that the water flux decreased with time at all applied pressures. The decrease in the water flux with time is due to membrane fouling and the concentration of the feed solution, as the reject solution was recycled back into the system. In addition, the reduction of the water flux could be due to the accumulation of organic matter on the surface of the membrane [17]. Figure 2a shows that in the RO process, the water flux at an applied pressure of 12–20 bar was within the same range, but at an applied pressure of 10 bar, the water flux was much lower. In the NF process, the water flux at an applied pressure of 12 bar was higher than the water flux in the other studied pressure values.

**Figure 3:**
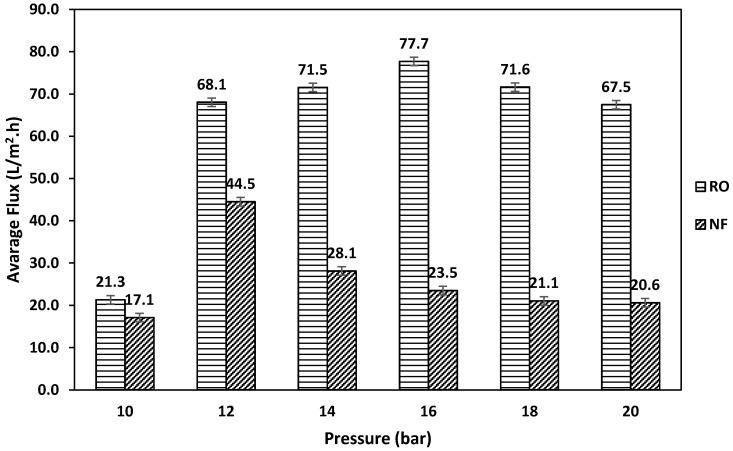
Average water flux of reverse osmosis (RO) and nanofiltration (NF) at different feed pressures.

Figure 3 shows the average water flux for RO and NF at the different studied feed pressures. In RO, the average water flux was 21.3 L/(m^2^·h) at a pressure of 10 bar. The average water flux increased by almost 69% to reach a value of 68.1 L/(m^2^·h) as the pressure increased to 12 bar. The average water flux reached a value of 71.5 L/(m^2^·h) when the applied pressure increased to 14 bar. The maximum water flux was 77.7 L/(m^2^·h), which was obtained at an applied pressure of 16 bar. As the applied pressure further increased, the average water flux decreased. The average water flux was 71.6 and 67.5 L/(m^2^·h) at a pressure of 18 and 20 bar, respectively. In the NF process, excluding the experiment with an applied pressure of 10 bar, it was found that the average water flux decreased as the pressure increased (Figure 3). The maximum average water flux was 44.5 L/(m^2^·h), which was obtained at an applied pressure of 12 bar. At an applied pressure of 14 bar, the average water flux decreased to almost 37%, reaching a value of 28.1 L/(m^2^·h). As the applied pressure further increased, the average water flux continued to decrease, reaching a minimum value of 20.6 L/(m^2^·h) at an applied pressure of 20 bar. The water permeability is expected to increase as the feed pressure increases; however, applying excessive pressure may result in excessive accumulation of foulants on the surface of the membrane, which may result in a lower average water flux [18]. The lowest average water flux for RO and NF was obtained at an applied pressure of 10 bar. This is due to the fact that the water diffusion through the membrane starts to occur when the applied pressure exceeds the natural osmotic pressure of the feed solution. It was found that the osmotic pressure of the feed solution was almost 9 bar; consequently, a low feed pressure of 10 bar was not enough to overcome the osmotic pressure of the feed solution. It can be observed that the water flux obtained using RO was higher than NF; this can be attributed to the fact that treated wastewater is rich in organic foulants. Therefore, the membrane fouling could be a dominant factor in this experiment. Although the larger pore size of the NF membrane can result in higher water flux, the foulants could penetrate through the membrane pores, unlike the RO membrane, causing a more severe fouling effect [19,20]. The contact angle of the NF 90 membrane was 72.2°, while that of BW30LE was 63.7°; therefore, the RO membrane was found to be more hydrophilic compared to the NF membrane. Enhanced hydrophilicity of the RO membrane means more strongly bounded water layer at the surface of the membrane, which may act as a barrier for foulants and hence reduce fouling [13,21]. The zeta potential of NF 90 was −60 mv, and that of BW30LE was −32 mv; the NF membrane has a higher negative charge when compared to the RO membrane. This makes the NF membrane more prone to fouling due to the high attraction force between the membrane and positively charged foulants [22,23]. The RMS roughness of the NF90 membrane was 61 nm, and that of BW30LE was 49.7 nm; the membrane with the rougher surface is more prone to fouling [24]. Similar results were obtained in previous studies—the NF90 membrane displayed severe fouling when used for wastewater treatment, which resulted in a lower level of water flux than RO [21,25]. A more detailed discussion is provided on membrane fouling to support the findings regarding water flux.

**Figure 4:**
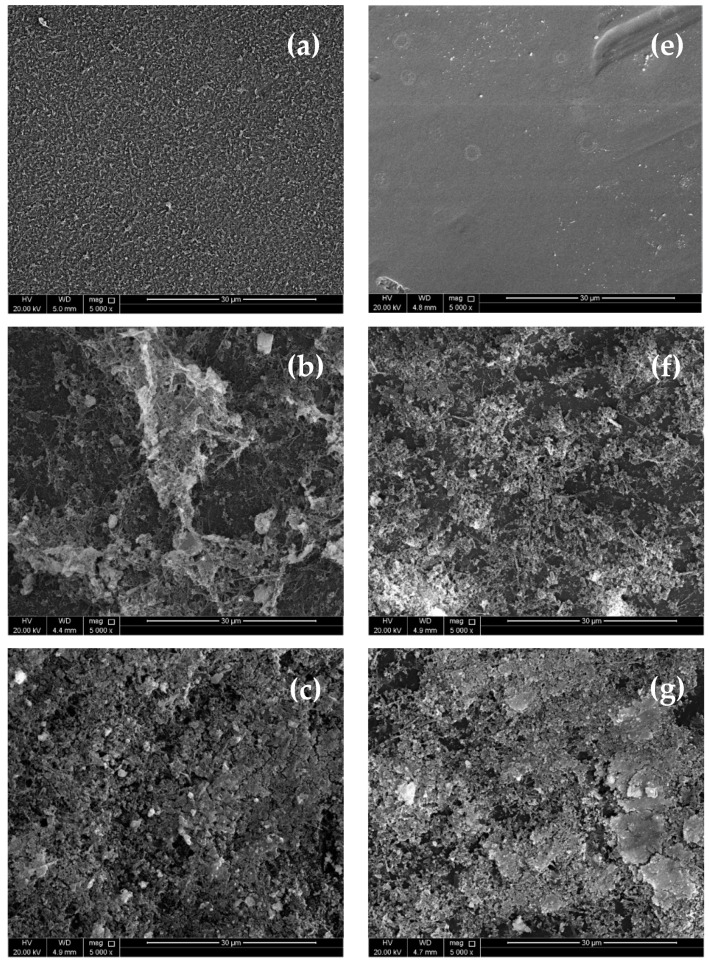

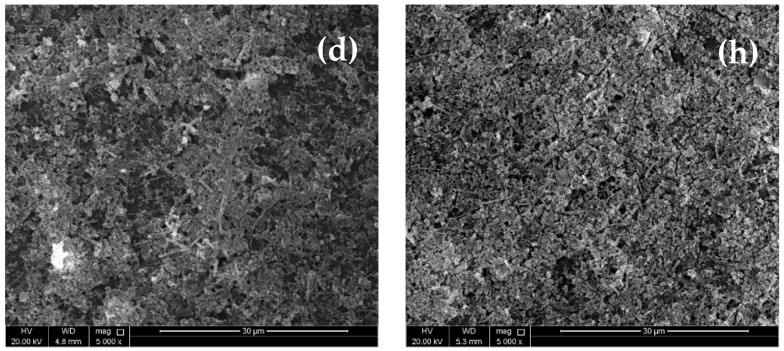
SEM images of (**a**) a clean RO membrane, (**b**) a tested RO membrane at a feed pressure of 12 bar, (**c**) a tested RO membrane at a feed pressure of 16 bar, (**d**) a tested RO membrane at a feed pressure of 20 bar, (**e**) a clean NF membrane, (**f**) a tested NF membrane at a feed pressure of 12 bar, (**g**) a tested NF membrane at a feed pressure of 16 bar, and (**h**) a tested NF membrane at a feed pressure of 20 bar.

SEM images of unused and used RO and NF membranes at different applied pressures are shown in Figure 4b–d. The used RO membranes at an applied pressure of 12, 16 and 20 bar can be observed. It can be seen from the SEM images that as the applied pressure increased, more accumulation of foulant materials occurred on the surface of the membrane. A similar observation was detected on the nanofiltration membrane, where the amount of the accumulated foulants increased on the surface of the membrane as the feed pressure increased (Figure 4f–h). TSE is rich in organic matter that could be attracted to the membrane surface by electrostatic forces. Moreover, studies showed that divalent ions such as calcium, which is attracted to the surface of the membrane by electrostatic forces, could bridge organic matters to the membrane surface, causing further fouling [26]. From the EDX analysis shown in Table 3, it can be seen that after the use of the RO and NF membranes, new elements were detected on the surface of the membranes, such as Na, Mg, Si, P, S, Cl, K, Ca, and Fe; this indicates the accumulation of these ions on the surface of the membrane. Table 3 shows that the amount of accumulated ions on the surface of the RO membrane was higher than that for the NF membrane. This indicates the higher rejection rate of these ions by RO when compared to NF.

### 3.2. Energy Consumption

**Figure 5:**
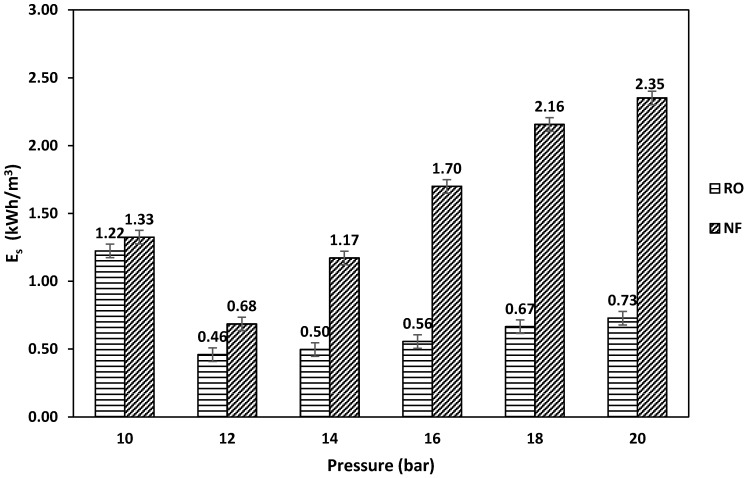
Specific energy consumption of RO and NF at different feed pressures.

Specific energy $\left(E_{s}\right)$ for RO and NF was calculated using Equation (2) [27]:

(2) $$E_{s}=\left(\frac{P}{n\;\times \;\%R}\right)$$

where *P* is pressure (bar), *n* is pump efficiency, and %*R* is recovery rate. From Equation (2), it can be seen that the specific energy depends on both the applied pressure and recovery rate, where the lowest specific energy will be obtained at a high recovery rate and low pressure. Figure 5 shows the specific energy consumption of the RO process and the NF process at different feed pressures. In the NF process, the specific energy consumption increased from 0.68 to 2.35 kWh/m^3^ at a feed pressure of 12 and 20 bar, respectively. As shown in Equation (2), the specific energy is a function of the applied pressure and recovery rate. At a low feed pressure of 10 bar, the specific energy consumption was 1.33 kWh/m^3^. The high specific energy consumption at such a low feed pressure is due to the low recovery rate obtained at a feed pressure of 10 bar (Figure 4). The maximum energy consumption was 2.35 kWh/m^3^, which was obtained at a feed pressure of 20 bar. The high specific energy consumption at such a high applied pressure is due to the low recovery. In the RO process, the same trend was observed, where the specific energy consumption increased from 0.46 to 0.73 kWh/m^3^ at a feed pressure of 12 and 20 bar, respectively. At a low feed pressure of 10 bar, the specific energy consumption was 1.22 kWh/m^3^, which is due to the low recovery rate obtained at such a low applied pressure. It was found that the NF process at an applied pressure of 12 bar gave the highest water recovery rate and the lowest energy consumption. For the RO process, the lowest energy consumption was found at an applied pressure of 12 bar, while the highest water recovery rate was at an applied pressure of 16 bar. The difference in the water recovery rate at an applied pressure between the 12 and 16 bar in the RO process was only 3.3%, while the energy consumption was 18% higher at an applied pressure of 16 bar when compared to an applied pressure of 12 bar. The quality of the produced permeate should be analyzed to investigate which process and which running conditions should be utilized.

### 3.3. Product Water Quality

**Figure 6:**
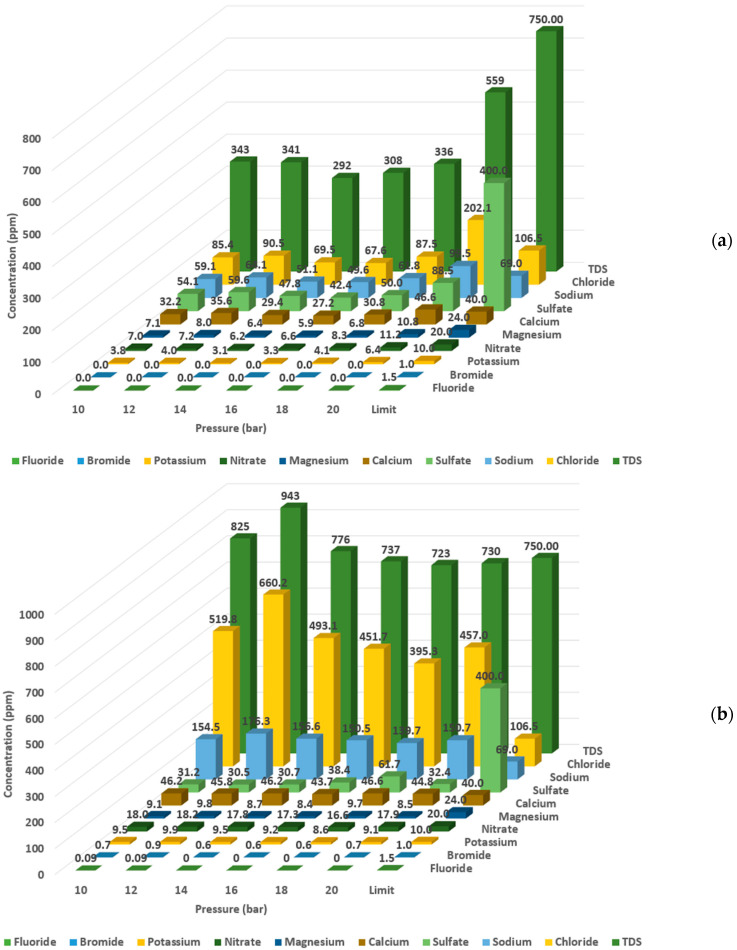
Characteristics of the permeate produced from treated sewage effluent (TSE) using (**a**) reverse osmosis (**b**) nanofiltration at different applied pressures.

Characteristics of the produced permeate were measured for the RO and the NF processes. The quality of the produced permeate was compared with the Food and Agriculture Organization (FAO) standards [12]. As shown in Figure 6a, the concentration of the different measured elements in the produced permeate from the RO process at applied pressures between 10 and 18 bar complied with the FAO standards. At an applied pressure of 20 bar, multiple parameters (such as TDS; conductivity; and chloride, sodium, and calcium concentration) exceeded the allowable limits. In the NF process (Figure 6b), under all applied pressures, the permeate quality did not comply with FAO standards. It was observed that the elements that did not comply with the standards were chloride, sodium and calcium. The high concentration of these elements in return affected the TDS concentration. For example, at an applied pressure of 12 bar where the highest water recovery rate was attained, the TDS concentration was almost 21% higher than the allowable limit, and the chloride, sodium and calcium concentrations were 84%, 61% and 13% higher than the allowable limit, respectively. It was observed that the NF membrane had a low rejection rate for monovalent ions, where the rejection rate for chloride and sodium was only 27% and 12%, respectively. The membrane rejection rate depends mainly on the molecular weight cut-off (MWCO); the MWCO of NF90 is 200–400 Da, and the MWCO of BW30LE is 100 Da. As the MWCO increases, the rejection rate decreases [28,29,30].

It can be inferred that using a single-stage NF is not possible due to the low water quality; thus, it is recommended to use RO at pressure applied between 10 and 18 bar. Selecting the most suitable applied pressure to operate the RO process depends on the water flux, the recovery rate, energy consumption and product water quality. The lowest energy consumption in RO was obtained at pressures applied between 12 and 14 bar. After considering the water quality, it is recommended to use an applied pressure of 14 bar due to the higher water quality.

## 4. Conclusions

In this paper, a comparative study was conducted on the reclamation of tertiary treated sewage effluent (TSE) by nanofiltration and reverse osmosis for water reuse in irrigation. It was found that reverse osmosis (RO) is suitable for the reclamation of tertiary treated sewage effluent (TSE) to be used as irrigation water for food crops. However, NF is not suitable for the reclamation of wastewater due to its low rejection rate for monovalent ions. In NF, the concentration of Na and Cl ions exceeded the maximum allowable limits recommended by FAO. In RO, the highest recovery rate was 36%, which was achieved at an applied pressure of 16 bar. The specific energy consumption at this applied pressure was 0.56 kWh/m^3^. At an applied pressure of 14 bar, the recovery rate was only 2% lower than that at an applied pressure of 16 bar, while the specific energy consumption was almost 11% lower. At an applied pressure of 12 bar, the specific energy consumption was 8% higher than the specific energy at an applied pressure of 14 bar, while the recovery rate was 7% higher. It is recommended to use the RO process at an applied pressure of 14 bar for the reclamation of TSE. This is due to the high recovery rate, low energy consumption and high water quality. In the future, the capital and operational costs of both processes must be compared at an industrial or pilot scale to ensure the economic feasibility of the process.

## Figures and Tables

**Table 1:** Characteristics of the feed water (treated municipal sewage effluent).

Parameter	Value	Max Limit (Irrigation Water)	Standard Testing Method
TDS (ppm)	1461 ± 5	750	APHA-2540 C/total dissolved solids dried at 180 °C
Turbidity (NTU)	0.2 ± 0.1	2	APHA-2130B/nephelometric
Electrical conductivity (mS/cm)	2.56 ± 0.2	0.7	APHA-2510B/conductivity
Dissolved organic carbon (ppm)	6.67 ± 0.05	-	Diaphragm electrode method
Fluoride (ppm)	0.27 ± 0.2	1.5	APHA-4110/determination of anions by ion chromatography
Chloride (ppm)	897.5 ± 0.2	106.5	
Bromide (ppm)	0.96 ± 0.2	1	
Nitrate (ppm)	25.84 ± 0.2	20	
Sulfate (ppm)	320.3 ± 0.2	400	
Sodium (ppm)	200.3 ± 0.2	69	APHA-3120/determination of metals by plasma emission spectroscopy
Potassium (ppm)	12.4 ± 0.2	10	
Calcium (ppm)	87.7 ± 0.2	40	
Magnesium (ppm)	21.4 ± 0.2	24	
Iron (ppm)	0.59 ± 0.02	5	ASTM D1068-15/standard test methods for iron in water
Boron (ppb)	158.97 ± 0.1	500	EPA method 200.8
Vanadium (ppb)	0.11 ± 0.1	100	
Manganese (ppb)	11.54 ± 0.1	200	
Cobalt (ppb)	0.17 ± 0.1	50	
Nickel (ppb)	23.11 ± 0.1	200	
Copper (ppb)	13.08 ± 0.1	200	
Zinc (ppb)	151.58 ± 0.1	2000	
Cadmium (ppb)	0.2 ± 0.1	10	
Beryllium (ppb)	2.02 ± 0.1	100	

**Table 2:** Characteristics for the nanofiltration and reverse osmosis membrane used in the experiment.

Properties	NF 90 [13]	BW30LE [14,15]
Contact angle	72.2°	63.7°
Zeta potential (mV)	−60	−32
Root mean square roughness (nm)	61	49.7
Molecular weight cut-off (Da)	200–400	100
Thickness (nm)	293	150

**Table 3:** Analysis of elements *wt*% on the surface of a clean and tested RO and NF membranes.

Membrane	Weight %										
	(C)	(O)	(Na)	(Mg)	(Si)	(P)	(S)	(Cl)	(K)	(Ca)	(Fe)
RO—clean	87.39	9.53	0	0	0	0	2.98	0	0	0	0
RO—tested	70.64	19.45	0.5	0.08	0.22	0.66	3.32	0.3	0.05	0.75	1.79
NF—clean	89.78	6.66	0	0	0	0	3.56	0	0	0	0
NF—tested	75.2	17.28	0.26	0.05	0.08	0.53	3.94	0.1	0	0.34	1.75

## Data Availability

All data generated or analyzed during this study are included in this published article.
